# The value of head imaging after PET-CT staging in NSCLC

**DOI:** 10.1186/1470-7330-15-S1-P16

**Published:** 2015-10-02

**Authors:** A Kamil, L Bashir, Y Griffin

**Affiliations:** 1 University Hospitals Of Leicester NHS Trust, Leicester, UK

## 

20-40% of patients with non small cell lung cancer (NSCLC) can develop brain metastases. Physiological activity on ^**18**^F- FDG PET-CT can mask FDG-avid brain metastases. At our institution PET-CT is acquired from orbits to thighs and will not identify brain metastases extrinsic to the posterior fossa. Inaccurate staging could initiate futile radical treatment.

The British Thoracic Society recommends NSCLC patients, considered for radical treatment (particularly stage III), should have a CT or MRI brain.

## Aim

We evaluated results of patients who underwent head imaging in those considered for radical treatment of NSCLC and effect on baseline ^**18**^F- FDG PET-CT staging.

## Method

Retrospective study. 200 NSCLC cases from 2010 to 2015 that underwent PET-CT for potentially curative disease, were reviewed.

## Results

17% (34/200) patients had head imaging. There was an average interval of 115 days between head imaging and PET-CT, ranging from 1 day to up to 2 years.

67% (23/34) patients received head imaging due to presenting neurological symptoms.

32% (15/49) patients with Stage IIIA disease had a CT/MRI brain resulting in 27% (4/15) having their initial PET-CT staging increased.

7.5% (15/200) patients had brain metastases. 93% (14/15) of these patients had presenting neurological symptoms.

## Conclusion

Occult brain metastases can cause under staging with ^**18**^F- FDG PET-CT imaging. CT/MRI head imaging requires more vigorous implementation into routine staging of patients with potentially resectable NSCLC.

